# Association of Initial Chest CT Findings, CT Severity Score and Clinical Parameters with ICU Admission in Hospitalized COVID-19 Patients

**DOI:** 10.3390/v18050528

**Published:** 2026-04-30

**Authors:** Aleksandra Milenkovic, Simon Nikolic, Jelena Aritonovic Pribakovic, Branislava Radovic, Aleksandra Ilic, Milica Stevanovic, Sara Kovacevic, Kristina Bulatovic, Jelena Milovanovic, Arijeta Kostic, Aleksandra Janicevic

**Affiliations:** 1 Faculty of Medicine in Priština, University of Priština Temporarily Settled in Kosovska Mitrovica, 38220 Kosovska Mitrovica, Serbia; simon.nikolic@med.pr.ac.rs (S.N.); jelena_km@hotmail.com (J.A.P.); tcmapple@gmail.com (B.R.); aleksandra.ilic@med.pr.ac.rs (A.I.); milicadstevanovic1@gmail.com (M.S.); sarakovkm@hotmail.com (S.K.); kristinajakovljevic@gmail.com (K.B.); jelena_krdzic@yahoo.com (J.M.); 2 Clinical Hospital Center Priština, 38205 Gracanica, Serbia; arijeta_pr@hotmail.com (A.K.); aleks121292@gmail.com (A.J.); 3 Clinical Hospital Center Kosovska Mitrovica, 38220 Kosovska Mitrovica, Serbia

**Keywords:** COVID-19, chest CT, severity score, predictors, intensive care unit

## Abstract

The aim of this study was to evaluate the association between baseline clinical and CT characteristics and to identify factors associated with intensive care unit (ICU) admission in hospitalized COVID-19 patients. This retrospective study included 176 adult patients with laboratory-confirmed SARS-CoV-2 infection hospitalized at the COVID Hospital of the Clinical Hospital Center Kosovska Mitrovica during 2021–2022 (Delta and Omicron variants). Patients were divided into two groups according to intensive care unit requirement: those treated in a general inpatient ward (No ICU) and those requiring ICU admission (ICU group). Demographic and clinical characteristics, lifestyle factors, CT findings, CT severity score (CTSS) values, and therapeutic interventions were compared between groups. Of the total cohort, 113 patients (64%) were hospitalized in a general inpatient ward, while 63 (36%) required intensive care unit admission. Independent predictors of ICU admission identified in the multivariate logistic regression analysis were obesity (B = 2.96, *p* < 0.001), dyspnea (B = 1.51, *p* = 0.041), higher CT severity score (B = 0.68, *p* < 0.001), and lower glucose levels (B = −0.27, *p* = 0.014). Furthermore, for each one-point increase in the CTSS, the odds of ICU admission nearly doubled (OR = 1.97). Total CT score values above the cut-off point (15.0) demonstrated significant reliability in discriminating the need for ICU transfer in patients with COVID-19. These findings suggest that combined clinical and radiological assessment at hospital admission may facilitate early identification of patients at high risk of requiring ICU care, with the CT severity score representing the strongest radiological predictor.

## 1. Introduction

An outbreak of a novel coronavirus (SARS-CoV-2), first detected in China in December 2019, subsequently evolved into a global public health concern and created considerable difficulties for healthcare systems worldwide. The substantial burden placed on healthcare facilities created new challenges in terms of work organization and the provision of appropriate diagnostic, therapeutic, and follow-up approaches for affected patients [1]. The consequences of the pandemic include not only the acute effects of infection but also long-term sequelae, such as post-COVID-19 syndrome and deterioration of mental health due to stress and social isolation.

COVID-19 is associated with a broad spectrum of clinical manifestations, ranging from mild symptoms such as fever, cough, and myalgia to severe pneumonia, acute respiratory distress, multi-organ failure, and death [2,3]. Although most patients remain asymptomatic or develop mild symptoms, 15.7–26.1% experience severe form of the disease that necessitates hospital admission and intensive monitoring [4,5]. Furthermore, an estimated 5–8% of infected patients require intensive care unit (ICU) admission and are at increased risk of mortality [6,7].

Multiple risk factors contributing to the progression of COVID-19 to a severe and critical stage have been identified, including older age, male gender, and comorbidities such as hypertension, diabetes, obesity, lung diseases, heart, liver, and kidney disease, immunodeficiency conditions, and malignancies. Additionally, socioeconomic status, diet, lifestyle, geographical differences, ethnicity, exposed viral load, treatment initiation day, and health care quality have all been reported to influence individual outcomes. Elevated inflammatory markers and chest imaging findings at admission are associated with severe pneumonia, ICU admission, and death [8,9,10].

During the pandemic, clinical presentation, laboratory parameters, and imaging findings were widely used for diagnostic work-up and prognostic stratification. Despite the identification of multiple predictors and the development of several scoring systems (e.g., CURB-65, qCSI, and BCRSS), a simple and widely applicable COVID-19-specific scoring model remains lacking [11].

According to World Health Organization (WHO) recommendations [12], imaging techniques are used in the diagnostic evaluation of patients when RT-PCR testing is unavailable or negative despite clinical suspicion of COVID-19. They are also employed for patient triage in conjunction with clinical and laboratory parameters, as well as for the assessment of treatment response and disease outcome in hospitalized patients. Chest computed tomography (CT) plays an important role in the initial assessment of COVID-19 patients and demonstrates a positive correlation with inflammatory markers, which may facilitate effective prediction of disease severity and the need for ICU admission [1,13].

Chest CT findings in COVID-19 are nonspecific but follow a characteristic temporal evolution. Early disease is typically characterized by bilateral ground-glass opacities, predominantly in peripheral and posterior lung regions, often involving the lower lobes. In the progressive phase, these opacities increase with interlobular septal thickening (“crazy paving” pattern), followed by consolidation in the peak stage. Later, lesions gradually regress, although residual fibrosis or traction bronchiectasis may persist [14,15,16,17,18,19,20,21,22,23].

In addition to characterizing parenchymal abnormalities, chest CT enables assessment of the extent of lung involvement, which may be used to predict disease outcome and the need for intensive care unit (ICU) admission. In this context, the CT severity score has been introduced as a semi-quantitative, objective, and reproducible method for assessing pulmonary involvement. One of the most widely used systems is the CT Severity Score Index (CTSS) proposed by Pan et al. [20], in which each lung lobe is scored according to the extent of involvement, yielding a total score ranging from 0 to 25 [20,24,25,26,27,28].

The aim of this study was to identify the early radiological and clinical predictors of ICU admission among COVID-19 patients. In addition, we aimed to characterize patients requiring ICU admission in our region, as limited data are available from this setting.

## 2. Materials and Methods

### 2.1. Ethical Approval

Ethical approval from the institutional review boards was obtained for this retrospective study (decision number 786, 2022).

### 2.2. Data Collection

This retrospective study included 176 consecutively hospitalized patients with laboratory-confirmed SARS-CoV-2 infection at the COVID Hospital of the Clinical Hospital Center Kosovska Mitrovica during 2021 and 2022 (Delta and Omicron Variants). Upon admission, each participant underwent CT examination at the Department of Radiology and Ultrasound Diagnostics. Patients under the age of 18, pregnant women, individuals with negative PCR test results, and those with incomplete medical records were excluded from the study.

Patient health data and hospital treatment outcomes were obtained from the hospital information system. The COVID-19 diagnosis was confirmed by a positive real-time reverse transcription polymerase chain reaction (RT-PCR, Novel Coronavirus (2019-nCoV) Nucleic Acid Diagnostic Kit, Sansure Biotech, Changsha, Hunan Province, People’s Republic of China). Upon admission, blood samples were collected from all patients to determine a complete blood count with leukocyte differential, biochemical parameters, and oxygen saturation.

Non-contrast-enhanced chest CT imaging was performed with a 16-slice CT scanner (Hitachi Eclos, Hitachi Medical Corporation, Chiyoda-ku, Tokyo, Japan) in a designated computer room. Appropriate personnel protection measures were applied, and both the equipment and the environment were disinfected. The patient was positioned supine and instructed to hold their breath during acquisition, which ranged from the thoracic inlet to the diaphragmatic level. All CT images were processed on dedicated diagnostic workstations and independently interpreted by two experienced radiologists. Cases with disagreement were resolved by consensus. Lung changes caused by SARS-CoV-2 were assessed in relation to the initial symptom onset in each patient. The type and distribution of CT findings, and their association with initial laboratory parameters, were systematically evaluated. Disease severity was quantified using the CTSS index and categorized as mild, moderate, or severe based on CTSS values.

All patients were managed in accordance with the official National Protocol of the Republic of Serbia for the treatment of COVID-19. According to the current criteria, ICU admission for COVID-19 patients is indicated for those who develop a moderate to severe form of the disease accompanied by deterioration of vital functions. It is also indicated for patients with severe to critical illness characterized by marked hypoxemia despite oxygen therapy, early development of acute respiratory distress syndrome (ARDS), and features of cytokine storm, requiring continuous monitoring of vital functions [29].

According to the requirement for ICU admission, patients were categorized into two groups: those hospitalized in a general inpatient ward without requiring intensive care, and those transferred to the ICU. We subsequently compared demographic and clinical characteristics, lifestyle habits, types and distribution of CT findings, CTSS values, as well as differences in the necessity of specific therapeutic interventions between these two groups.

### 2.3. Statistical Analysis

All statistical tests were two-sided, with a significance level of α = 0.05. Analyses were performed using SPSS Statistics version 22 (IBM Corp., Armonk, NY, USA).

For descriptive statistical analysis, continuous variables were presented as mean ± standard deviation or as median with interquartile range (Q1–Q3), depending on the distribution normality, which was assessed using the Shapiro–Wilk test. Categorical variables were expressed as absolute and relative frequencies. Differences in means of numerical variables were tested using either the independent samples *t*-test or the Mann–Whitney U test, depending on data distribution. Differences in categorical variables were evaluated using the Chi-square test or Fisher’s exact test, as appropriate. To align the number of variables in the model with the sample size, independent variables were grouped into three multivariable models. All variables that were statistically significant in the univariate analysis at the level of *p* < 0.01 were included in one of three multivariable logistic regression models, with ICU admission as the dependent variable. Before including the variables in any of the models, multicollinearity was assessed, and no significant correlations were found among the independent variables. Model I included demographic characteristics, lifestyle factors, comorbidities, hospitalization data, and clinical symptoms, as well as age, which has been identified as a significant predictor of admission to the intensive care unit in similar studies [8,9,10]. Model II included laboratory parameters, with multicollinearity assessed a priori. Model III included computed tomography findings. Predictors that remained significant at the level of *p* < 0.05 within each model were subsequently entered into the final multivariable model. The predictive value of the total CTSS for ICU admission was evaluated using receiver operating characteristic (ROC) curve analysis. The area under the curve (AUC) with 95% confidence intervals (CI) was calculated, with sensitivity and specificity determined at various thresholds. The optimal cut-off value was defined as the point maximizing the Youden index (J = sensitivity + specificity − 1), representing the best balance between sensitivity and specificity.

## 3. Results

This study included 176 patients, of whom 113 (64%) were hospitalized in a general inpatient ward (No ICU), and 63 (36%) required admission to the intensive care unit (ICU). Patients who required admission to the intensive care unit (ICU) were significantly older (median age 68 [IQR 57–73] vs. 61 [IQR 48–70]; *p* = 0.023), were more likely to be smokers (*p* < 0.001), and had higher prevalence of hypertension (*p* < 0.001), cardiovascular disease (*p* = 0.005), diabetes mellitus (*p* = 0.003), chronic obstructive pulmonary disease (COPD) (*p* = 0.007), and obesity (*p* < 0.001). Unvaccinated patients were also significantly more likely to require ICU admission (*p* = 0.041) (Table 1).

Differences were also observed between the two groups in terms of COVID-19-related clinical presentations on admission, as shown in Table 2. Patients who required admission to the ICU had a significantly longer hospital stay (*p* < 0.01), while a statistically significantly higher number of discharged patients was observed in the non-ICU group (*p* < 0.01). Dyspnea (*p* = 0.04) and myalgia/arthralgia (*p* = 0.019) were more prevalent in the ICU-admitted group, as was elevated body temperature (*p* = 0.07).

The differences in the laboratory findings between the two groups were also significant. Leukocytosis (*p* = 0.038), neutrophilia (*p* = 0.001), lymphopenia (*p* < 0.001), higher CRP (*p* = 0.002), D-dimer (*p* = 0.036), AST (*p* = 0.019), LDH (*p* = 0.002), urea (*p* = 0.004) and glucose (*p* = 0.001) were more frequent in ICU-admitted patients on admission. On the other hand, this group had significantly lower levels of albumin (*p* = 0.001) and total protein (*p* = 0.001), as well as lower oxygen saturation (SPO_2_) values (*p* < 0.001) (Table 3).

Lung changes caused by SARS-CoV-2, diagnosed by chest CT, were carefully evaluated for each patient in relation to the onset of initial symptoms with the aim of illustrating their temporal evolution. (Supplementary Materials S1).

The distribution and type of CT changes, as well as the CTSS values, are presented in detail in Table 4 and show a statistically significant difference between two groups. In a significantly higher number of patients from the ICU group, lung changes involved upper lobes (*p* < 0.001) and right middle lobe (*p* = 0.005). Additionally, in these patients, the changes were distributed both centrally and peripherally (*p* > 0.001), affecting anterior and posterior regions of the lungs (*p* > 0.001). Pulmonary CT changes significantly associated with ICU admission included septal thickening (*p* = 0.002), crazy paving pattern (*p* = 0.041), consolidations (*p* < 0.001), subpleural bands (*p* = 0.003) and dilated pulmonary vessels (*p* < 0.022).

**Figure 1:**
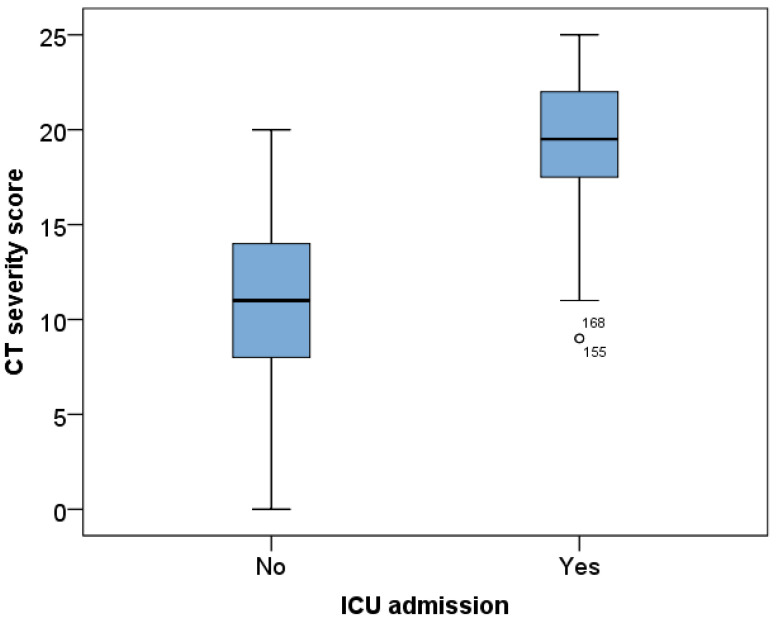
Distribution of total CTSS between ICU and non-ICU groups.

Higher CTSS was also significantly more prevalent among patients who required ICU admission (*p* < 0.001) (Figure 1).

All variables that were significant in the univariate analysis at the level of *p* < 0.01 were included in a multivariable logistic regression model with the dependent variable defined as ICU admission.

The final multivariable regression model included 12 predictors and was statistically significant (chi-square = 148.37, *p* < 0.001). Independent predictors of ICU admission identified in the multivariate logistic regression analysis were obesity (B = 2.96, *p* < 0.001), dyspnea (B = 1.51, *p* = 0.041), higher CT severity score (B = 0.68, *p* < 0.001), and lower glucose levels (B = −0.27, *p* = 0.014). A predictor associated with a reduced risk of admission to the intensive care unit was localization in the right middle lobe (RML) (B = −2.78, *p* = 0.041). Furthermore, for each one-point increase in the CTSS, the odds of ICU admission nearly doubled (OR = 1.97) (Table 5).

### ROC Analysis for Predicting the Requirement for ICU Admission

**Figure 2:**
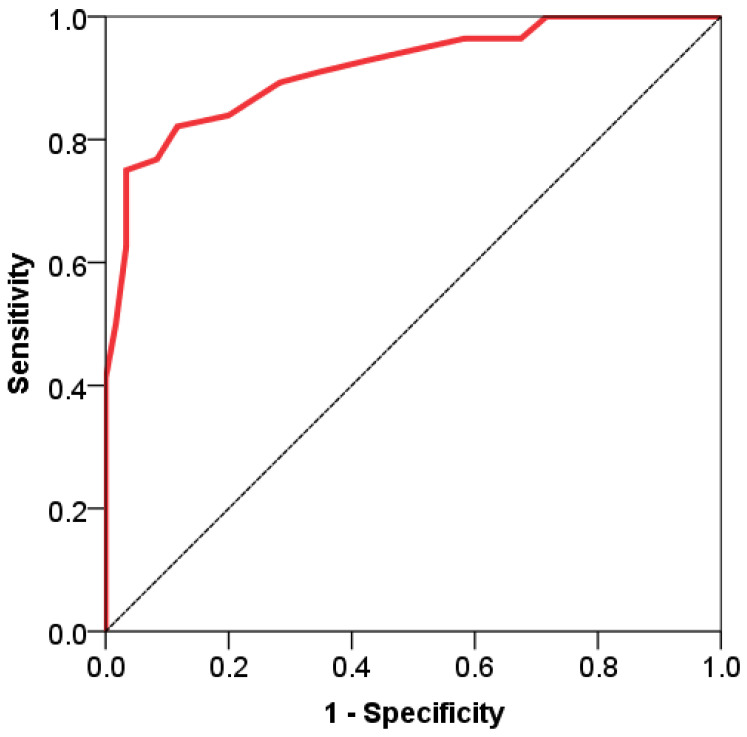
ROC curve of the total CT score for predicting the need for ICU transfer.

The total CTSS values were analyzed to predict the need for ICU admission using ROC analysis. The total CTSS proved to be strong discriminative criterion for determining the need for patient transfer to ICU. The area under the ROC curve (AUC) for the total CTSS was 0.933 (95% confidence interval, 0.885–0.965; *p* < 0.001). Sensitivity and specificity were 81.0% and 92.0%, respectively, and the Youden index was 0.73. Total CTSS values above the cut-off point (15.0) demonstrated significant reliability in discriminating the need for ICU transfer in patients with COVID-19 (Figure 2).

The association between statistically significant CT findings and laboratory parameters is presented in the Supplementary Materials S2.

## 4. Discussion

The aim of this study was to document the association between baseline clinical and CT characteristics and to identify factors influencing patient admission to ICU in order to facilitate clinical implementation of COVID-19 management and improve patient prognosis. We considered that predicting COVID-19 severity is crucial for managing general ward and ICU capacity, particularly in centers with limited admission and treatment resources such as ours.

This study identified obesity, dyspnea, smoking history, arterial hypertension, and body temperature as significant predictors and risk factors for ICU admission. Among laboratory parameters, glucose levels and SPO_2_ were important indicators of disease severity. With respect to radiological findings, right middle lobe involvement and CT severity score were statistically significantly associated with ICU admission in hospitalized COVID-19 patients. In particular, obesity, dyspnea, glucose levels, and CT severity score values were independent risk factors for ICU transfer in our study population, while right middle lobe localization showed a protective effect.

Numerous studies have indicated that smoking is linked to greater COVID-19 severity and higher mortality. Chronic exposure to cigarette smoke may elevate ACE2 receptor expression in the respiratory epithelium, potentially leading to adverse clinical outcomes. These findings are consistent with reports that active smoking is related to ICU admission, more severe disease form, and increased mortality [30,31,32], which aligns with our results. In contrast, Ainsworth et al. suggest that smoking is associated with ICU transfer in combination with other factors, including demographic, socioeconomic, and environmental characteristics, health-related risk factors, and behavioral practices [33].

Previous studies have shown that patients with comorbidities are more likely to develop severe clinical manifestations. Comorbid conditions such as arterial hypertension, cardiovascular and respiratory diseases, diabetes mellitus, obesity, and others heighten individual susceptibility and contribute to the development of more severe forms of COVID-19, potentially requiring ICU admission [2,5,6,7,8,24]. Our results are consistent with several studies that have demonstrated a strong association between obesity and ICU transfer. Obesity is thought to be driven by chronic inflammation, impaired immune function, and a predisposition to thrombosis, all of which contribute to a more severe clinical course. In addition, the detrimental effects of excess abdominal adipose tissue on respiration, elevated ACE2 receptor expression on adipocytes, impaired liver function, inadequate vitamin D levels, and insulin resistance further exacerbate disease severity [7,23,34]. Although arterial hypertension was also a significant comorbidity in our study, obesity demonstrated a stronger and more consistent association with ICU admission compared to arterial hypertension, which lost statistical significance in the adjusted model, suggesting that its effect may be mediated by other clinical factors. In their study, Abuyousef et al. [7] reported cardiovascular disease and diabetes as independent risk factors for ICU admission. The discrepancy with our findings may be explained by differences in sample size and the demographic, clinical, and other characteristics of the study population.

Dyspnea and SPO_2_ both reflect respiratory impairment. In COVID-19 patients, the severity of hypoxemia, reflected by lower SPO_2_ values, is independently associated with in-hospital mortality and may serve as a key predictor of ICU admission [35,36,37]. However, in our study, dyspnea remained a more consistent independent predictor of ICU admission, suggesting that subjective respiratory distress may better capture clinical deterioration than isolated oxygen saturation values. This discrepancy may be explained by the fact that SPO_2_ represents a single-point physiological measurement of oxygenation, which may be influenced by compensatory mechanisms or oxygen supplementation, whereas dyspnea reflects a more complex and integrated clinical manifestation of respiratory distress. Consequently, dyspnea may better capture the overall severity of illness and impending clinical deterioration in COVID-19 patients.

A growing body of literature has identified that poor glycemic control was associated with worse COVID-19 outcomes [38,39,40]. In the present study, glucose levels measured at hospital admission emerged as an independent laboratory predictor of ICU admission, with lower glucose levels being associated with increased odds of critical illness. This suggests that altered metabolic status at presentation may reflect more severe systemic disease and physiological stress response in COVID-19 patients. Although hyperglycemia has more commonly been reported as a marker of poor outcomes in COVID-19, our findings indicate that lower admission glucose levels may also be associated with disease severity, potentially reflecting impaired metabolic reserve or dysregulated host response in critically ill patients.

As previously mentioned, chest CT plays an important role in determining disease extent, monitoring progression, and assessing COVID-19 severity, thereby influencing the timeliness and effectiveness of treatment in hospitalized patients. Due to its lower specificity when compared with RT-PCR testing, CT is primarily used as an adjunctive diagnostic tool [15,17,40,41,42,43,44]. Currently, numerous prognostic tools in use employ clinical data to assess the risk of developing severe COVID-19 and the need for ICU transfer. Based on the obtained CT scans, we analyzed the types and distribution of radiological findings in relation to disease onset, as well as their association with laboratory parameters, as presented in the Supplementary Materials. By calculating CTSS, we quantified the extent of lung parenchymal involvement. The obtained CT findings were compared between non-ICU and ICU groups. Significant differences were observed between the two groups, with ICU patients showing greater upper lung lobe involvement and more extensive lesion distribution than the patients hospitalized in the general ward. This aligns with previously reported trends in COVID-19 imaging, where bilateral lung changes reflect more extensive disease, correlating with greater clinical severity and a higher likelihood of progression [41,42,43,44,45]. Our study more frequently documented the presence of septal thickening, subpleural bands, and consolidation in patients with more severe disease forms requiring ICU admission. This finding can be explained by the more pronounced inflammation of the lung parenchyma in patients with severe forms of the disease [46], resulting in a more complex pattern of chest CT abnormalities.

The CTSS was the only independent radiological predictor in the multivariate analysis, underscoring that the overall extent of pulmonary involvement has a greater prognostic value than specific lobar localization or individual imaging features. Overall, quantitative assessment of CT disease burden appears more clinically relevant than isolated radiological patterns in estimating ICU admission. ROC analysis demonstrated that a total CTSS > 15 has strong discriminative ability for predicting ICU transfer in hospitalized COVID-19 patients, suggesting its potential value as a practical imaging-based triage tool. Several studies have similarly confirmed the prognostic importance of CT-derived quantification of pulmonary involvement in COVID-19. Stasiow B. et al. reported that the extent of inflammatory changes on chest CT serves as an independent predictor of ICU admission, need for mechanical ventilation, and mortality, with a proposed CTSS threshold of 12, while scores above 15 were associated with fatal outcomes [47]. Similarly, Gorecki A. et al. identified CTSS values ≥ 13 as predictive of ICU admission [48], whereas Szabo et al. reported a higher threshold of 18.5 for ICU transfer [13]. These results differ from ours, likely due to multiple contributing factors. Additionally, differences in sample size and the time periods in which the studies were conducted during the pandemic—potentially influenced by the predominance of different viral variants—should also be considered.

## 5. Conclusions

In hospitalized COVID-19 patients, a combination of clinical, laboratory, and radiological parameters can effectively predict the risk of ICU admission. Independent predictors in our cohort included obesity, dyspnea, and admission glucose levels. Radiological extent of lung involvement was a stronger determinant of ICU admission than specific imaging patterns, with CTSS emerging as the strongest radiological predictor. In our cohort, a CTSS threshold > 15 demonstrated strong discriminatory performance for ICU admission, supporting its potential clinical utility in early risk stratification. This finding suggests that CT-based quantification of lung involvement may assist in identifying patients at higher risk of clinical deterioration at the time of hospital admission. However, CTSS should be interpreted in conjunction with clinical and laboratory parameters, rather than as a standalone triage tool, to support timely and comprehensive clinical decision-making.

Our research has several limitations. First, it is a retrospective study with a relatively small sample size. Due to limited institutional resources, genomic sequencing of SARS-CoV-2 variants was not available; therefore, variant attribution was based on epidemiological data on circulating variant predominance in Serbia during the study period, which may have led to potential misclassification. The low number of vaccinated patients in our cohort limited our ability to analyze the impact of vaccination on the outcomes. We are aware that ICU capacity and resources in our center are limited, which may have influenced admission decisions and potential bias. Although our CTSS cutoff was not externally validated, it was compared with published data and found to be generally consistent. Moreover, there was no opportunity to follow up on the condition of the survivors after hospital discharge. Finally, as a single-center study, our findings are limited to a specific geographic region, which may reduce their generalizability.

## Figures and Tables

**Table 1:** Main demographic characteristics, habits, and comorbidities of patients according to the ICU admission.

Parameters	ICU Admission		*p*
	No n = 113	Yes n = 63	
	n (%)	n (%)	
Gender			
Male	74 (65.5)	45 (71.4)	0.419
Female	39 (34.5)	18 (28.6)	
Age	61 (48–70)	68 (57–73)	0.023 *
Smoking history			
No	69 (61.1)	13 (20.6)	
Yes	20 (17.7)	31 (49.2)	<0.001 *
Former smoker	24 (21.2)	19 (30.2)	
Comorbidities			
Arterial hypertension	61 (54.0)	52 (82.5)	<0.001 *
Cardiovascular diseases	20 (17.7)	23 (36.5)	0.005 *
Diabetes mellitus	20 (17.7)	24 (38.1)	0.003 *
COPD	0 (0.0)	4 (6.3)	0.007 *
Neurological diseases	7 (6.2)	3 (4.8)	0.694
Psychiatric diseases	2 (1.8)	4 (6.3)	0.109
Malignant conditions	8 (7.1)	4 (6.3)	0.854
Obesity	22 (19.5)	32 (50.8)	<0.001 *
Vaccination status			
Unvaccinated	73 (64.6)	50 (79.4)	0.041 *
Vaccinated	40 (35.4)	13 (20.6)	
Vaccination			
Incomplete	11 (27.5)	5 (38.5)	0.455
Complete	29 (72.5)	8 (61.5)	

Numerical data are presented as median (Q1–Q1), and categorical data are presented as number of patients and percentage, n (%). * Statistically significant value; Abbreviation: ICU—Intensive Care Unit.

**Table 2:** Clinical characteristics of patients.

Parameters	ICU Admission		*p*
	No n = 113	Yes n = 63	
	n (%)	n (%)	
Duration of symptoms before admission (days)	7 (4–11.5)	8 (6–14)	0.113
Duration of hospital stay (days)	12 (9–16)	18 (10–26)	<0.001 *
Disease outcome			
Discharged	108 (95.6)	12 (19)	<0.001 *
Died	5 (4.4)	51 (81)	
Symptoms			
Fever	105 (92.9)	59 (93.7)	0.854
Cough	63 (55.8)	39 (61.9)	0.428
Dyspnea	36 (31.9)	34 (54.0)	0.004 *
Sore throat	6 (5.3)	5 (7.9)	0.490
Chest tightness	13 (11.5)	12 (19.0)	0.169
Runny nose	4 (3.5)	1 (1.6)	0.455
Fatigue	83 (73.5)	50 (79.4)	0.381
Headache	21 (18.6)	9 (14.3)	0.467
Gastrointestinal symptoms	37 (32.7)	17 (27.0)	0.427
Anosmia	10 (8.8)	3 (4.8)	0.320
Myalgia/arthralgia	26 (23.0)	25 (39.7)	0.019 *
Body temperature (°C)	37.8 (37.4–38.2)	38 (37.6–39)	0.007 *

Numerical data are presented as median (Q1–Q1), and categorical data are presented as number of patients and percentage, n (%). * Statistically significant value.

**Table 3:** Initial laboratory parameters stratified by ICU admission.

Parameters	ICU Admission		*p*
	No n = 113	Yes n = 63	
	n (%)	n (%)	
Leukocytes (×10^9^/L)	7.2 (5.2–11.1)	9.2 (6.3–14)	0.038 *
Neutrophils, (%)	0.8 (0.7–0.86)	0.8 (0.8–0.9)	0.001 *
Lymphocytes, (%)	0.1 (0.08–0.2)	0.1 (0.05–0.1)	<0.001 *
Erythrocytes (×10^12^/L)	4.5 (4.0–4.7)	4.5 (4.0–5.0)	0.268
Hemoglobin (g/L)	130 (120–139)	132 (119–147)	0.323
Platelets (×10^9^/L)	209 (154–281)	182 (139–279)	0.211
CRP, (mg/L)	69 (25–127)	106 (65–159)	0.002 *
D-dimer (ng/mL)	800 (329–2120)	1350 (770–2230)	0.036 *
Fibrinogen (g/L)	5.8 (4.8–6.6)	6.2 (4.6–7.2)	0.223
AST (U/L)	33 (27–50)	44 (29–70)	0.019 *
ALT (U/L)	35 (25–52)	40 (28–62)	0.117
LDH (U/L)	455 (358–577)	541 (432–726)	0.002 *
Albumin (g/L)	37 (32–41)	33 (30–36)	0.001 *
Total proteins (g/L)	68 (61–71)	63 (58–68)	0.001 *
Glucose (mmol/L)	6.7 (5.7–8.6)	8.8 (6.1–13)	0.001 *
Creatinine (µmol/L)	81 (69–102)	84 (72–109)	0.313
Urea (mmol/L)	6.1 (4.4–8.3)	7.1 (5.7–10.4)	0.004 *
SPO_2_ (%)	95 (91–97)	88 (87–90)	<0.001 *

Numerical data are presented as median (Q1–Q1), and categorical data are presented as number of patients and percentage, n (%). * Statistically significant value. Abbreviations: CRP: C-reactive protein; AST: aspartate aminotransferase; ALT: alanine aminotransferase; LDH: lactate dehydrogenase; SPO_2_: oxygen saturation. Note: Reference ranges for laboratory parameters are as follows: Leukocytes 4.0–10.0 × 10^9^/L, Neutrophils 40–70%, Lymphocytes 20–40%, Erythrocytes 4.1–5.9 × 10^12^/L, Hemoglobin 120–175, Platelets 150–400 × 10^9^/L, CRP < 5 mg/L, D-dimer < 0.5 mg/L, Fibrinogen 2.0–4.0 g/L, AST 10–40 U/L; ALT 7–56 U/L, LDH 140–280 U/L, Albumin 35–50 g/L, Total proteins 60–80 g/L, Glucose 3.9–5.5 mmol/L, Creatinine 44–106 µmol/L, Urea 2.5–7.5 mmol/L, SPO_2_ 95–100%.

**Table 4:** Initial chest computed tomography findings stratified by ICU admission.

Parameters	ICU Admission		*p*
	No n = 113	Yes n = 63	
	n (%)	n (%)	
Distribution of CT finding			
Unilateral	9 (8.0)	2 (3.2)	0.113
Bilateral	96 (85.0)	60 (95.2)	
No abnormalities	8 (7.1)	1 (1.6)	
Localization RUL	32 (28.3)	43 (68.3)	<0.001 *
Localization RML	93 (82.3)	61 (96.8)	0.005 *
Localization RLL	107 (95)	62 (98.4)	0.226
Localization LUL	70 (61.9)	56 (88.9)	<0.001 *
Localization LLL	103 (91.2)	62 (98.4)	0.056
Axial distribution			
Central	1 (0.9)	0 (0.0)	
Peripheral	77 (69.4)	11 (17.5)	
Central and peripheral	33 (29.7)	52 (82.5)	<0.001 *
Anteroposterior distribution			
Anterior	2 (1.8)	0 (0.0)	
Posterior	76 (68.5)	9 (14.3)	
Anterior and posterior	33 (29.7)	54 (85.7)	<0.001 *
Type of CT changes			
Groundglass opacities	94 (83.2)	51 (81.0)	0.709
Septal thickening	53 (46.9)	45 (71.4)	0.002 *
Crazy paving	50 (44.2)	38 (60.3)	0.041 *
Consolidation	70 (61.9)	61 (96.8)	<0.001 *
Reverse halo sign	16 (14.2)	12 (19.0)	0.395
Subpleural bands	47 (41.6)	41 (65.1)	0.003 *
Dilated pulmonary vessels	84 (74.3)	56 (88.9)	0.022 *
Pulmonary fibrosis	35 (31)	19 (30.2)	0.911
Traction bronchiectasis	23 (20.4)	19 (30.2)	0.143
Pleural effusion	25 (22.1)	18 (28.6)	0.340
Pneumothorax	3 (2.7)	5 (7.9)	0.107
Lymphadenopathy	14 (12.4)	6 (9.5)	0.566

Categorical data are presented as number of patients and percentage, n (%); * Statistically significant value. Abbreviations: CT—computed tomography; RUL—right upper lobe; RML—right middle lobe; RLL—right lower lobe; LUL—left upper lobe; LLL—left lower lobe.

**Table 5:** A multivariate logistic regression analysis with ICU admission as dependent variable.

Independent Variables	Parameters					
	B	*p*	OR (95% CI)	B	*p*	OR (95% CI)
**Model 1.**						
Age	0.01	0.536	1.01 (0.98–1.05)			
Smoking history						
No	reference category			reference category		
Yes	**1.22**	**0.014**	**3.38 (1.28–8.94)**	0.37	0.665	6.02 (1.01–35.88)
Former smoker	**1.49**	**0.004**	**4.46 (1.61–12.36)**	1.66	0.073	5.26 (0.86–32.35)
Arterial hypertension	**1.16**	**0.033**	**3.18 (1.10–9.19)**	0.95	0.213	2.58 (0.58–11.47)
Cardiovascular d.	0.36	0.444	1.43 (0.57–3.57)			
Diabetes Mellitus	0.28	0.551	1.32 (0.53–3.26)			
COPD	21.34	0.999	1.8 × 10^9^ (0.0–)			
Obesity	**1.90**	**<0.001**	**6.69 (2.82–15.88)**	**2.96**	**0.001**	**19.21 (3.61–102.21)**
Dyspnea	**0.98**	**0.021**	**2.68 (1.16–6.19)**	**1.51**	**0.041**	**4.54 (1.07–19.37)**
Body temperature	**0.59**	**0.045**	**1.81 (1.01–3.24)**	0.01	0.983	1.01 (0.43–2.36)
**Model 2.**						
Neutrophils	4.47	0.170	87.35 (0.15–51,863.0)			
Lymphocytes	−7.54	0.125	0.001 (0.00–8.14)			
CRP	−0.002	0.519	1.00 (0.99–1.00)			
LDH	**0.002**	**0.004**	**1.00 (1.00–1.00)**	0.001	0.199	1.00 (1.00–1.00)
Albumin	−0.03	0.558	0.97 (0.89–1.07)			
Total proteins	**−0.06**	**0.032**	**0.94 (0.88–1.00)**	−0.07	0.071	0.93 (0.87–1.01)
Glucose	**−0.15**	**0.024**	**0.86 (0.76–0.98)**	**−0.27**	**0.014**	**0.76 (0.62–0.95)**
Urea	−0.03	0.450	0.97 (0.89–1.06)			
SPO_2_	**−0.309**	**<0.001**	**0.73 (0.65–0.83)**	−0.08	0.330	0.92 (0.79–1.08)
**Model 3.**						
Localization RUL	−0.58	0.382	0.56 (0.15–2.06)			
Localization RML	**−3.88**	**0.002**	**0.02 (0.0–0.25)**	**−2.78**	**0.041**	**0.06 (0.00–0.90)**
Localization LUL	**−1.88**	**0.029**	**0.15 (0.03–0.83)**	−0.92	0.380	0.40 (0.05–3.10)
Axial distribution						
Central	reference category					
Peripheral	4.93	1.00	137.9 (0.0–)			
Cent. and Peripher.	5.23	1.00	187.6 (0.0–)			
Anteroposterior distribution						
Anterior	reference category					
Posterior	11.30	1.00	8.1 × 10^4^ (0.0–)			
Ant. i Post.	12.38	1.00	2.3 × 10^5^ (0.0–)			
CT severity score	**0.72**	**<0.001**	**2.06 (1.55–2.74)**	**0.68**	**<0.001**	**1.97 (1.45–2.68)**
Septal thickening	0.89	0.194	2.45 (0.64–9.44)			
Consolidation	1.04	0.278	2.83 (0.43–18.60)			
Subpleural bands	−0.13	0.845	0.88 (0.24–3.17)			

B—beta coefficient, OR—odds ratio, CI—confidence interval, Bolded—statistically significant predictor (*p* < 0.05).

## Data Availability

The data presented in this study are available on request from the corresponding author.

## Supplementary Materials

The following supporting information can be downloaded at: https://www.mdpi.com/article/10.3390/v18050528/s1, Table S1: CT Findings According to Symptom Duration Prior to Admission; Figure S1: SPO2 values according to the presence of septal thickening; Table S2: Laboratory parameter values according to the presence of consolidations; Table S3: Laboratory parameter values according to the presence of subpleural bands.
