# Supplementary material for: Association of Initial Chest CT Findings, CT Severity Score and Clinical Parameters with ICU Admission in Hospitalized COVID-19 Patients
Source: Viruses. 2026 Apr 30;18(5):528. doi: 10.3390/v18050528 (PMC13211654; doi:10.3390/v18050528)
Supplement: Supplementary file 1 [file viruses-18-00528-s001.zip › viruses-4274980-supplementary.pdf]

Supplementary Material S1.

The chest CT findings of a total of 176 patients with COVID-19 pneumonia were analyzed in relation to the duration of symptoms prior to hospital admission. Symptom duration was divided into four intervals: 0–4 days, 5–8 days, 9–13 days, and ≥14 days. The frequency of all CT findings was evaluated within these predefined time intervals. Ground-glass opacities (GGO) were most frequent in the early stages of symptom onset, observed in 95.1% of patients within 0–4 days and 89.3% within 5–8 days. The frequency of GGO decreased with longer symptom duration prior to admission but remained prevalent in patients with symptoms lasting 9–13 days (73.2%) and ≥14 days (68.4%) ($p = 0.003$). Interlobular septal thickening and the crazy paving pattern showed significant differences according to symptom duration before admission (both $p < 0.001$). These changes were most frequent in the 5–8 days interval (87.5% for both). Consolidation also demonstrated significant variation with symptom duration ($p < 0.001$), peaking between 9–13 days (90.2%). The reversed halo sign showed statistically significant differences according to symptom duration ($p = 0.003$), although overall prevalence was low, with the highest frequency observed in the 5–8 days interval (28.6%). Subpleural bands were significantly more frequent ($p < 0.001$) in patients with longer symptom duration (9–13 days and ≥14 days: 78.0% and 78.9%, respectively). The prevalence of fibrotic changes and traction bronchiectasis also varied significantly with symptom duration ($p < 0.001$ for both). In patients admitted ≥14 days after symptom onset, all patients (100.0%) had fibrosis, and 86.8% exhibited bronchiectasis (Supplementary material 1).

Table S1. CT Findings According to Symptom Duration Prior to Admission

**Types of CT changes number (%)**	**Duration of symptoms prior to admission (days)**				***P***
	**0-4**	**5-8**	**9-13**	**≥14**	
	41 (23.3)	56 (31.8)	41 (23.3)	38 (21.6)	
Groundglass opacities	39 (95.1)	50 (89.3)	30 (73.2)	26 (68.4)	0.003*
Septal thickening	7 (17.1)	49 (87.5)	30 (73.2)	12 (31.6)	<0.001*
Crazy paving	7 (17.1)	49 (87.5)	29 (70.7)	3 (7.9)	<0.001*
Consolidation	19 (46.3)	44 (78.6)	37 (90.2)	31 (81.6)	<0.001*
Reverse halo sign	6 (14.6)	16 (28.6)	6 (14.6)	0 (0.0)	0.003*
Subpleural bands	2 (4.9)	24 (42.9)	32 (78.0)	30 (78.9)	<0.001*
Dilated pulmonary vessels	31 (75.6)	48 (85.7)	35 (85.4)	26 (68.4)	0.142
Pulmonary fibrosis	0 (0.00)	1 (1.8)	15 (36.6)	38 (100. 0)	<0.001*
Traction bronchiectasis	0 (0.0)	2 (3.6)	7 (17.1)	33 (86.8)	<0.001*
Pleural effusion	8 (19.5)	13 (23.2)	11 (26.8)	11 (28.9)	0.770
Pneumothorax	0 (0.0)	2 (3.6)	2 (4.9)	4 (10.5)	
PTE	1 (2.4)	5 (8.9)	5 (12.2)	1 (2.6)	
Lymphadenopathy	4 (9.8)	6 (10.7)	3 (7.3)	7 (18.4)	0.445
Other (nodules, bullous changes)	0 (0.0)	1 (1.8)	3 (7.3)	5 (13.2)	

Categorical data are presented as number of patients and percentage, n (%); *Statistically significant value.

Supplementary Material S2.

Laboratory values were analyzed with respect to the presence of septal thickening in the lungs diagnosed by chest CT, and oxygen saturation (%) emerged as a statistically significant parameter ($p = 0.002$). Oxygen saturation values were statistically significantly higher in patients without CT-diagnosed septal thickening in the lungs (93.0 ± 4.3% vs. 90.5 ± 5.7%), as shown in figure.

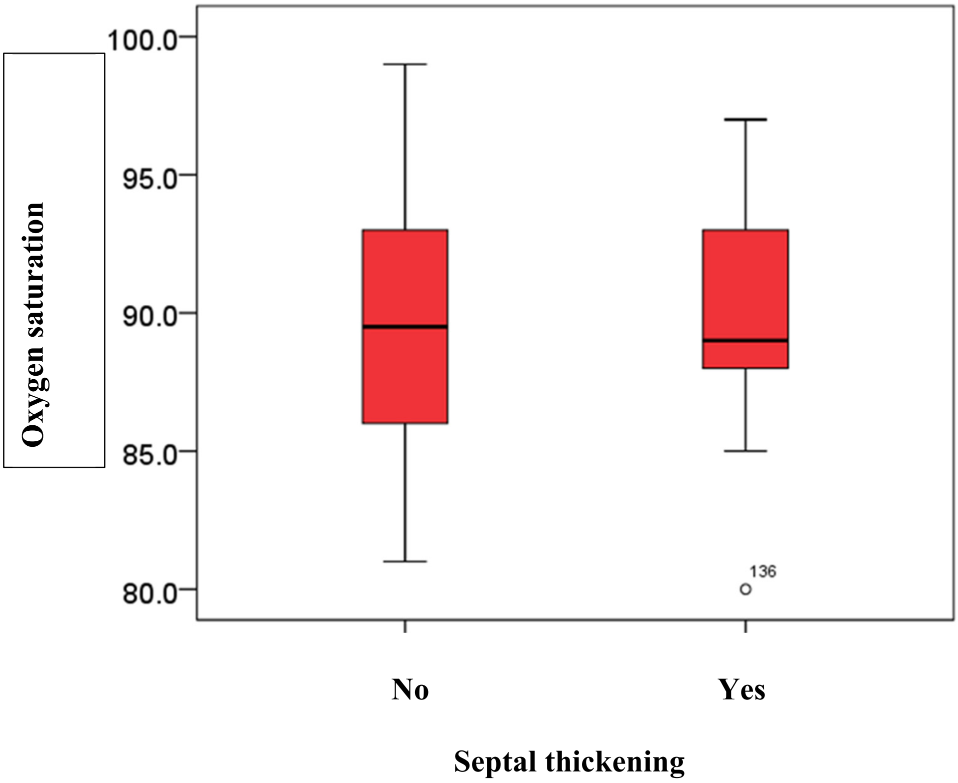

Figure S1. SPO_2 values according to the presence of septal thickening

By analyzing differences in laboratory parameter values between patients with and without CT-diagnosed pulmonary consolidations, ten parameters were identified as statistically significant (Supplementary material 3). Statistically significantly higher values in patients without consolidations were observed for oxygen saturation ($p < 0.001$; 95.0 ± 3.2 vs. 90.4 ± 5.4), lymphocytes ($p < 0.001$; 0.2 ± 0.1 vs. 0.1 ± 0.06), albumin ($p < 0.001$; 39.0 [35.0–42.5] vs. 34.0 [30.0–38.0]), and total protein ($p < 0.001$; 68.0 [63.0–73.0] vs. 64.0 [59.0–69.0]). Laboratory parameters with statistically significantly higher values in patients with diagnosed consolidations included neutrophils ($p < 0.001$; 0.7 ± 0.1 vs. 0.8 ± 0.1), C-reactive protein (CRP) ($p < 0.001$; 38.0 [14.5–117.0] vs. 91.0 [52.0–153.0]), fibrinogen ($p = 0.003$; 5.3 ± 1.5 vs. 6.2 ± 1.8), AST ($p = 0.001$; 31.0 [24.0–38.5] vs. 42.5 [29.0–63.0]), ALT ($p = 0.028$; 32.0 [19.5–47.0] vs. 38.0 [28.0–61.0]), and glucose ($p < 0.004$; 7.1 ± 2.5 vs. 9.0 ± 4.2).

Table S2. Laboratory parameter values according to the presence of consolidations

**Parameters**	**Consolidation**		***P***
	**No**	**Yes**	
Total number (%)	45 (25.6%)	131 (74.4%)	
SPO_2 (%)	95.0±3.2	90.4±5.4	<0.001*
Leukocytes, (x10^9/L)	7.7±4.3	11.1±16.9	0.184
Neutrophils, (%)	0.7±0.1	0.8±0.1	<0.001*
Lymphocytes, (%)	0.2±0.1	0.1±0.06	<0.001*
Erythrocytes (x10^{12}/L)	4.4±0.6	4.4±0.7	0.561
Hemoglobin (g/L)	128.7±18.6	129.8±19.3	0.725
Platelets, 10^9/l	223.8±92.6	215.8±95.5	0.626
CRP, (IU/ml)	38.0 (14.5-117.0)	91.0 (52.0-153.0)	<0.001*
D-dimer (ng/ml)	698.0 (346.0-1868.5)	1210.0 (485.0-2300.0)	0.138
Fibrinogen (g/L)	5.3±1.5	6.2±1.8	0.003*
AST (U/L)	31.0 (24.0-38.5)	42.5 (29.0-63.0)	0.001*
ALT (U/L)	32.0 (19.5-47.0)	38.0 (28.0-61.0)	0.028*
LDH (U/L)	502.9±260.8	550.9±257.9	0.833
Albumin (g/L)	39.0 (35.0-42.5)	34.0 (30.0-38.0)	<0.001*
Total proteins (g/L)	68.0 (63.0-73.0)	64.0 (59.0-69.0)	<0.001*
Glucose (mmol/L)	7.1±2.5	9.0±4.2	0.004*
Creatinine (mmol/L)	80.0 (74.0-102.0)	84.0 (69.0-106.0)	0.995
Urea (mmol/L)	6.3 (4.1-7.8)	6.8 (4.9-9.6)	0.145

Laboratory parameters are presented as mean ± standard deviation or median (Q1–Q3), depending on the normality of the data distribution. Categorical data are presented as number of patients and percentage, n (%); * Statistically significant value. Abbreviations: SPO_2: oxygen saturation; CRP: C reactive protein; AST: aspartate aminotransferase; ALT: alanine aminotransferase; LDH: lactate dehydrogenase.

In patients with CT-diagnosed subpleural bands in the lungs, C-reactive protein (CRP) values were statistically significantly higher ($p < 0.001$; 77.9 ± 68.9 vs. 126.9 ± 92.4), as were fibrinogen ($p = 0.024$; 5.5 [4.6–6.6] vs. 6.2 [5.0–7.2]), AST ($p = 0.024$; 33.5 [26.0–49.0] vs. 42.0 [28.2–63.0]), and LDH ($p = 0.001$; 441.5 [356.5–532.8] vs. 545.5 [393.0–681.5]). Patients without subpleural bands had statistically significantly higher oxygen saturation (SpO_2) values ($p = 0.006$; 92.7 ± 4.4 vs. 90.5 ± 5.8)

Table S3. Laboratory parameter values according to the presence of subpleural bands

**Parameters**	**Subpleural bands**		***P***
	**No**	**Yes**	
Total number (%)	88 (50.0%)	88 (50.0%)	
SPO_2 (%)	92.7±4.4	90.5±5.8	0.006*
Leukocytes, (x10^9/L)	9.2±5.8	11.4±20.1	0.317
Neutrophils, (%)	0.8±0.1	0.8±0.1	0.279
Lymphocytes, (%)	0.1±0.08	0.1±0.9	0.265
Erythrocytes (x10^{12}/L)	4.4±0.7	4.5±0.6	0.590
Hemoglobin (g/L)	129.5±19.0	129.5±19.2	0.994
Platelets, 10^9/l	214.3±87.7	221.4±101.4	0.623
CRP, (IU/ml)	77.9±68.9	126.9±92.4	<0.001*
D-dimer (ng/ml)	852.0 (392.5-1987.5)	1355.0 (527.5-2715.0)	0.055
Fibrinogen (g/L)	5.5 (4.6-6.6)	6.2 (5.0-7.2)	0.024*
AST (U/L)	33.5 (26.0-49.0)	42.0 (28.2-63.0)	0.024*
ALT (U/L)	37.0 (25.0-48.8)	38.0 (26.0-62.0)	0.223
LDH (U/L)	441.5 (356.5-532.8)	545.5 (393.0-681.5)	0.001*
Albumin (g/L)	36.0 (30.2-41.0)	35.0 (31.0-37.8)	0.177
Total proteins (g/L)	65.1±7.8	63.6±8.7	0.222
Glucose (mmol/L)	7.0 (5.8-9.1)	7.5 (5.9-11.3)	0.401
Creatinine (mmol/L)	80.0 (71.0-96.8)	84.0 (69.8-119.2)	0.122
Urea (mmol/L)	7.2±4.8	8.5±4.9	0.071

Laboratory parameters are presented as mean ± standard deviation or median (Q1–Q3), depending on the normality of the data distribution. Categorical data are presented as number of patients and percentage, n (%); * Statistically significant value. Abbreviations: SPO_2: oxygen saturation; CRP: C reactive protein; AST: aspartate aminotransferase; ALT: alanine aminotransferase; LDH: lactate dehydrogenase.
